# A Genetic and Biochemical Perspective: Acute Coronary Syndrome and Raftlin

**DOI:** 10.3390/jcm15124400

**Published:** 2026-06-06

**Authors:** Rıdvan Bora, Burak Toprak, Emrah Yeşil, Rojda Tanrıverdi, Muhammed Adıyaman, Mustafa Demir, Oben Döven

**Affiliations:** 1 Department of Cardiology, Mersin City Education and Research Hospital, 33240 Mersin, Turkey; dr.ridvanbora@outlook.com; 2 Department of Cardiovascular Surgery, Mersin City Education and Research Hospital, 33120 Mersin, Turkey; 3 Department of Cardiology, Mersin University Faculty of Medicine Hospital, 33343 Mersin, Turkey; emrhyesil@gmail.com (E.Y.); obendoven@icloud.com (O.D.); 4 Department of Biochemistry, Mersin University Faculty of Medicine Hospital, 33110 Mersin, Turkey; rjd_tnv_73@hotmail.com; 5 Department of Cardiology, Private Genesis Hospital, 21070 Diyarbakır, Turkey; dr.madiyaman@gmail.com; 6 Department of Cardiology, Defne State Hospital, 31160 Hatay, Turkey; drmustafademirr@gmail.com

**Keywords:** acute coronary syndrome, atherosclerosis, raftlin, gene polymorphism, inflammation

## Abstract

**Background:** Inflammation plays a central role in the pathophysiology of acute coronary syndrome (ACS). Raftlin, a lipid raft-associated inflammatory protein involved in immune signaling, has emerged as a potential biomarker in cardiovascular disease. This study aimed to evaluate circulating raftlin levels and RFTN1 rs690037 polymorphism in patients with ACS. **Methods:** This prospective observational study included 100 participants comprising 50 patients diagnosed with ACS and 50 control subjects with angiographically normal coronary arteries. Serum raftlin concentrations were measured using enzyme-linked immunosorbent assay, and RFTN1 rs690037 polymorphisms were analyzed by real-time polymerase chain reaction. Correlation, receiver operating characteristic (ROC), multivariable logistic regression, and net reclassification improvement (NRI) analyses were performed. **Results:** Serum raftlin levels were significantly higher in the ACS group compared with controls (4.28 [3.48–5.78] vs. 3.38 [2.66–4.23] ng/dL, *p* = 0.003). Raftlin levels demonstrated significant positive correlations with LDL cholesterol and HbA1c levels in ACS patients (*p* < 0.05 for both). ROC analysis showed that raftlin had moderate discriminative ability for ACS detection (AUC: 0.672, 95% CI: 0.570–0.762, *p* = 0.002). Although raftlin was not independently associated with ACS after multivariable adjustment, incorporation of raftlin into the baseline clinical model improved overall risk classification (NRI: 0.213, *p* = 0.041). No significant association was observed between RFTN1 rs690037 polymorphism and ACS or circulating raftlin levels. **Conclusions:** Circulating raftlin levels are elevated in patients with ACS and appear to reflect the inflammatory and metabolic dysregulation accompanying acute coronary events. Although raftlin alone demonstrated limited diagnostic performance, its incremental contribution to multimarker risk assessment models suggests potential utility as a complementary inflammatory biomarker in ACS. Larger multicenter studies are warranted to clarify its prognostic and translational significance.

## 1. Introduction

Cardiovascular disorders continue to represent the most common cause of mortality on a global scale, with ischemic heart disease constituting a substantial proportion of cardiovascular-related deaths [1]. Data reported by the World Health Organization indicate that cardiovascular diseases were responsible for approximately 31% of total worldwide mortality in 2020 [2]. A significant proportion of these deaths are attributed to acute coronary syndrome (ACS). According to the 2012 European Cardiovascular Disease Statistics, cardiovascular diseases accounted for 45% of mortality in women and 38% in men younger than 75 years of age [3].

Acute coronary syndrome is a clinical condition characterized by myocardial ischemia and associated symptoms resulting from plaque rupture, vasospasm, or an imbalance between oxygen supply and demand in the coronary arteries. Acute coronary syndrome encompasses a spectrum of clinical presentations resulting from acute myocardial ischemia, including ST-segment elevation myocardial infarction (STEMI), non-ST-segment elevation myocardial infarction (NSTEMI), and unstable angina.

Atherosclerosis is a chronic inflammatory disease of the arterial wall and represents the principal pathological substrate underlying most cases of acute coronary syndrome (ACS). The development of atherosclerosis is strongly associated with traditional cardiovascular risk factors, including smoking, diabetes mellitus, hypertension, dyslipidemia, obesity, and aging. These factors contribute to endothelial dysfunction, lipid accumulation within the vascular intima, oxidative stress, and recruitment of inflammatory cells into the arterial wall. Progressive inflammatory activation promotes formation and progression of atherosclerotic plaques through interactions among endothelial cells, macrophages, smooth muscle cells, and circulating lipoproteins. In ACS, the critical event is typically plaque rupture or plaque erosion followed by thrombus formation and acute coronary artery obstruction rather than atherosclerosis alone. Therefore, inflammatory activity plays a central role not only in plaque progression but also in plaque destabilization and thrombotic complications leading to acute coronary event [4].

Following endothelial damage, mechanical, hormonal, and hemodynamic changes occur. While the endothelium normally promotes vasodilation and anticoagulation, injury transforms it into a procoagulant, proatherogenic, and proinflammatory state. Additionally, LDL particles present in the environment act as chemotactic agents for inflammatory cells. Inflammation plays a central role in the weakening of plaques, contributing to plaque rupture and acute coronary events [5]. Endothelial injury and oxidized LDL particles play a crucial role in sustaining inflammation and contributing to the development of ACS [5,6].

Lipid rafts are specialized microdomains within the plasma membrane, enriched with glycosphingolipids, cholesterol, and protein receptors, which play a pivotal role in cellular signaling, endocytosis, and disease processes. They are involved in various cellular processes, including signal transduction, endocytosis, exocytosis, and the entry of intracellular pathogens. It has been proposed that lipid rafts play a critical role in signaling by facilitating interactions between receptors and effectors involved in signal transmission [7]. Raftlin, a significant protein in lipid rafts, has been associated with various inflammatory diseases, including atherosclerosis, highlighting its potential as a biomarker in ACS [7]. It should be acknowledged that raftlin is not a disease-specific biomarker and may be elevated in various inflammatory and immune-mediated conditions. Therefore, its potential clinical value in acute coronary syndrome should be interpreted within the broader inflammatory context rather than as a standalone diagnostic marker. Given its multifaceted role in immune signaling and endothelial activation, raftlin presents a compelling target for investigation in diseases marked by chronic inflammation, such as ACS. Recent findings have demonstrated that raftlin may also participate in endothelial cell activation and vascular inflammation, particularly through modulation of IL-6 and NF-κB pathways [8].

Raftlin is one of the major lipid raft proteins and is believed to play a significant role in B-cell signaling and activation. Additionally, raftlin modulates T-cell receptor signaling. It has been reported that raftlin is involved in inducing Th17-mediated autoimmune responses and vascular inflammatory responses [9,10].

To our knowledge, limited data are available regarding the combined evaluation of ACS, circulating raftlin levels, and RFTN1 gene polymorphism. Despite growing interest in lipid raft-associated proteins, the clinical utility of raftlin in cardiovascular diseases remains largely unexplored. The aim of this study was to investigate the relationship between circulating raftlin levels and acute coronary syndrome (ACS) and to evaluate the potential association between raftlin-related inflammatory activity and clinical characteristics of ACS patients. The primary endpoint of the study was the difference in circulating serum raftlin levels between patients with ACS and control participants with angiographically normal coronary arteries. Secondary endpoints included the evaluation of associations between raftlin levels and selected clinical and biochemical parameters, including LDL cholesterol, HbA1c, CRP, troponin, CK-MB, renal function markers, and left ventricular ejection fraction. Additional secondary analyses included assessment of the discriminative performance of raftlin for ACS detection using receiver operating characteristic (ROC) analysis and investigation of the relationship between RFTN1 rs690037 genotype distribution, circulating raftlin levels, and ACS status through comparative genotype and allele frequency analyses. Finally, the study explored whether raftlin may provide complementary inflammatory and metabolic information when interpreted together with established clinical and biochemical markers in ACS.

## 2. Materials and Methods

- **Data Collection**

**Study Design:**

This prospective study was conducted at the Cardiology Department of Mersin University Faculty of Medicine between February 2023 and January 2024. The study was designed and reported in accordance with the Strengthening the Reporting of Observational Studies in Epidemiology (STROBE) guidelines for observational studies. A total of 100 participants were included, comprising 50 patients diagnosed with acute coronary syndrome (ACS) who were hospitalized and 50 individuals in the control group who were followed up and treated in the cardiology outpatient clinic. Eligible participants were enrolled consecutively during the study period in order to reduce potential selection bias. The control group consisted of individuals with no history of coronary artery disease. All participants in the control group underwent conventional angiography or coronary computed tomography angiography within the last six months. Only individuals with normal coronary arteries on imaging were included in the study. Patients aged ≥18 years who were diagnosed with ACS according to current guideline-based clinical, electrocardiographic, and biochemical criteria were eligible for inclusion in the ACS group. The ACS cohort included patients presenting with ST-segment elevation myocardial infarction (STEMI), non-ST-segment elevation myocardial infarction (NSTEMI), and unstable angina according to contemporary guideline definitions. Smoking status was defined based on active cigarette smoking at the time of study enrollment. Former smokers who had quit smoking prior to enrollment were not classified as active smokers in the statistical analyses. Patients with active infection, autoimmune or chronic inflammatory disease, malignancy, severe hepatic failure, advanced renal failure requiring dialysis, recent major surgery or trauma, known hematological disorders, spontaneous coronary artery dissection, or insufficient clinical/laboratory data were excluded from the study. These exclusion criteria were applied to minimize the influence of major systemic inflammatory conditions and severe comorbid states that could independently alter circulating inflammatory biomarker levels, including raftlin. In particular, chronic inflammatory diseases such as rheumatoid arthritis and advanced renal failure requiring dialysis are associated with persistent immune activation, endothelial dysfunction, and accelerated atherosclerosis, all of which may substantially affect inflammatory signaling pathways independent of ACS itself. Therefore, exclusion of these patient groups was intended to reduce potential biological confounding and to allow a more specific evaluation of the relationship between ACS and circulating raftlin levels. Nevertheless, this approach may limit the generalizability of the findings to broader real-world cardiovascular populations with complex inflammatory comorbidities. Similar exclusion criteria were also applied to the control group to minimize potential confounding inflammatory conditions. However, although angiographically normal coronary anatomy was confirmed, these individuals may not fully represent the general healthy population because they had undergone cardiovascular evaluation due to clinical suspicion or symptoms requiring diagnostic imaging. Importantly, the absence of obstructive epicardial coronary artery disease does not completely exclude the presence of coronary microvascular dysfunction or subclinical endothelial inflammatory activity. Therefore, it is possible that some control participants may have had underlying microvascular coronary disease or low-grade vascular inflammation that was not directly assessed in the present study. This potential overlap may have attenuated intergroup differences in circulating inflammatory biomarkers, including raftlin, and should be considered a possible source of selection bias when interpreting the findings. The required sample size was calculated using an assumed effect size of 0.80, a Type I error rate of 1%, and a statistical power of 90%. This effect size was based on preliminary data suggesting a mean difference in serum raftlin levels of 0.90 ng/dL between ACS and control subjects, with a standard deviation of approximately 1.2 ng/dL. This calculation indicated a requirement of at least 43 participants per group. To compensate for possible data attrition, the number of participants was expanded to 50 in each group, yielding a total study population of 100 individuals.

Sample size estimations were performed with the G*Power software package (version 3.1.9.4).

**Data Collection:**

The analysis of serum raftlin levels and raftlin gene (rs690037) polymorphism was conducted at the Department of Medical Biochemistry, Mersin University Hospital. Blood samples were collected from participants into two types of tubes: EDTA-containing tubes for genetic analysis and plain biochemistry tubes for serum analysis. In patients with ACS, blood samples were obtained during the initial hospital evaluation prior to invasive intervention whenever clinically feasible. However, detailed information regarding prior statin exposure, chronic antiplatelet therapy, and exact timing of pharmacological treatment initiation before blood sampling was not systematically standardized or incorporated into the statistical analyses. For serum raftlin analysis, blood collected in plain tubes was centrifuged at 4000 rpm for 10 min to obtain serum samples. Separated sera were preserved at −80 °C until analysis.

Quantification of raftlin concentrations was performed using an enzyme-linked immunosorbent assay (ELISA). Serum raftlin levels were measured using a commercially available human raftlin ELISA kit according to the manufacturer’s instructions. The assay detection range, intra-assay coefficient of variation, and inter-assay coefficient of variation were evaluated in accordance with the kit protocol to ensure analytical reliability. The intra-assay and inter-assay coefficients of variation were below 10%, indicating acceptable analytical reproducibility. Sample concentrations were calculated based on optical density (OD) values obtained from standard curves. Genetic analysis involved blood samples collected in EDTA-containing tubes, which were stored at +4 °C. DNA was isolated from these samples using standard isolation procedures. The raftlin gene (rs690037) polymorphisms were then analyzed using Real-Time PCR technology, ensuring precise genotyping and reliable results. Genotyping reactions were performed using allele-specific probes under standardized thermal cycling conditions according to the manufacturer’s protocol. To ensure genotyping accuracy, randomly selected samples were reanalyzed, and concordance between repeated analyses was confirmed. This approach provided comprehensive data on both biochemical and genetic parameters, ensuring the reliability and scientific integrity of the study findings. Routine biochemical and hematological parameters, including CRP, troponin, CK-MB, lipid profile, renal function markers, glucose-related parameters, and complete blood count variables, were analyzed in the central hospital laboratory using standardized automated laboratory methods routinely applied in daily clinical practice. All laboratory analyses were performed in accordance with institutional quality control protocols.

b. **Data Analysis**

**Statistical Analysis:**

Data normality was evaluated separately in the ACS and control groups using the Shapiro–Wilk test. Continuous variables with normal distribution were expressed as mean ± standard deviation, whereas non-normally distributed variables were presented as median with interquartile range (IQR). Categorical variables were summarized as frequencies and percentages.

Comparisons between the ACS and control groups were performed according to variable type and distribution characteristics. Student’s *t*-test was used for normally distributed continuous variables, while the Mann–Whitney U test was applied for non-normally distributed continuous variables. Categorical variables were compared using the chi-square test. When appropriate, *p*-values below 0.05 were considered statistically significant.

Correlation analyses were performed to evaluate the relationship between serum raftlin levels and clinical, biochemical, and inflammatory parameters. Pearson correlation analysis was used for normally distributed variables, whereas Spearman correlation analysis was preferred for variables that did not meet normality assumptions. Correlation coefficients were interpreted together with their statistical significance, and weak correlations were cautiously evaluated in the context of the exploratory nature of the study.

Receiver operating characteristic (ROC) curve analysis was performed to assess the discriminative performance of serum raftlin levels for distinguishing patients with ACS from control subjects. The area under the curve (AUC), 95% confidence interval (CI), optimal cut-off value, sensitivity, and specificity were calculated. Additional comparative ROC analyses were performed for conventional biomarkers, including CRP, troponin, and CK-MB. A baseline clinical model was constructed using clinically relevant inflammatory, metabolic, and demographic variables, including CRP, LDL cholesterol, HbA1c, left ventricular ejection fraction (LVEF), smoking status, age, and sex. Subsequently, serum raftlin levels were added to this baseline model to evaluate their incremental contribution to ACS risk discrimination and reclassification analyses.

Multivariable logistic regression analysis was performed to determine whether raftlin was independently associated with ACS after adjustment for potential confounding variables. Given the relatively limited sample size, the number of variables included in the multivariable model was intentionally restricted in order to reduce the risk of model overfitting and to maintain an acceptable events-per-variable ratio. Variables included in the model were selected based on clinical relevance, baseline intergroup differences, and their potential confounding effect on the relationship between raftlin and ACS. Results were reported as odds ratios (ORs) with 95% confidence intervals and *p*-values. Model calibration was additionally assessed using the Hosmer–Lemeshow goodness-of-fit test.

The distribution of RFTN1 rs690037 genotypes in the ACS and control groups was assessed using the Hardy–Weinberg equilibrium test. Genotype frequencies were compared between groups, and odds ratios with 95% confidence intervals were calculated to evaluate possible associations between genotype distribution and ACS status. Raftlin levels were also compared across genotype subgroups.

Net reclassification improvement (NRI) analysis was performed to evaluate whether the addition of raftlin improved ACS risk classification beyond the baseline clinical model. Positive NRI values were interpreted as improved reclassification after adding raftlin to the model. However, no formal internal validation method such as bootstrap resampling or cross-validation was performed because of the exploratory nature and limited sample size of the study. To further evaluate model stability and potential optimism, bootstrap resampling was performed to estimate optimism-corrected AUC values and confidence intervals for ΔAUC after the addition of raftlin. In addition, 5-fold cross-validation analyses were applied to assess the internal stability of the baseline clinical model and the baseline clinical model plus raftlin.

Given the exploratory nature of the study and the relatively limited sample size, formal correction for multiple comparisons was not routinely applied. Therefore, statistically significant findings, particularly weak correlation results, were interpreted cautiously and considered hypothesis-generating rather than confirmatory.

**Software:**

Statistical analyses were performed using IBM SPSS version 21 and MedCalc software version 22.030. Normality assumptions were evaluated using the Shapiro–Wilk test prior to statistical analyses. Parametric or non-parametric tests were selected according to data distribution characteristics. Cases with missing key laboratory or clinical variables were excluded from the final statistical analysis, and no imputation method was applied because the proportion of missing data was minimal.

## 3. Results

The data in Table 1 demonstrates that the mean age of patients in the ACS group was 58.20 years (±6.94), while it was 57.40 years (±5.52) in the control group, with no statistically significant difference between the groups (*p* = 0.525). The gender distribution was also comparable, with 48% female and 52% male in the ACS group, and 52% female and 48% male in the control group (*p* = 0.689). The mean BMI was 27.41 (±3.95) in the ACS group and 28.39 (±3.53) in the control group, without significant difference (*p* = 0.198). However, a significant difference was observed in left ventricular ejection fraction (EF), with a median EF of 50 [40–55] in the ACS group and 60 [60–60] in the control group (*p* < 0.0001). The prevalence of diabetes mellitus (DM) was 34% in the ACS group and 28% in controls (*p* = 0.517), while hypertension (HT) was observed in 42% and 58% of patients in the ACS and control groups, respectively (*p* = 0.110). Hyperlipidemia (HL) was equally distributed (22% in both groups, *p* = 1.00). Notably, smoking prevalence was significantly higher in the ACS group (70%) compared to the control group (46%) (*p* = 0.015). Serum raftlin levels were also significantly elevated in the ACS group, with a median of 4.28 ng/dL [3.48–5.78], compared to 3.38 ng/dL [2.66–4.23] in the control group (*p* = 0.003) (Table 1).

The correlation analysis presented in Table 2 revealed that in the total population, raftlin levels were positively correlated with LDL levels (r = 0.223, *p* = 0.025) and HbA1c (r = 0.201, *p* = 0.045), both reaching statistical significance. In the ACS group, these correlations were even more pronounced, with raftlin levels significantly associated with LDL (r = 0.298, *p* = 0.036) and HbA1c (r = 0.335, *p* = 0.017). However, no statistically significant correlations were identified in the control group across any of the evaluated variables (*p* > 0.05). The remaining variables, including age, BMI, urea, creatinine, GFR, sodium, potassium, uric acid, HDL, triglycerides, BNP, troponin, CK-MB, LVEF, and CRP, showed no significant correlations with raftlin levels in either the total population or within the subgroup analyses (Table 2).

According to Table 3, the ROC analysis revealed that raftlin levels demonstrated a statistically significant ability to differentiate between ACS patients and controls, with an area under the curve (AUC) of 0.672 (95% CI: 0.570–0.762; *p* = 0.0020). The optimal cut-off value for raftlin was determined to be >3.8324 ng/dL, which provided a sensitivity of 68% (95% CI: 53.3–80.5) and a specificity of 68% (95% CI: 53.3–80.5). These findings indicate that raftlin has moderate diagnostic performance as a biomarker for ACS detection (Table 3).

According to Table 4, genotype analysis demonstrated that the TT genotype was observed in 18.0% of the ACS group and 24.0% of the control group, serving as the reference category. The TC genotype was present in 50.0% of ACS patients and 42.0% of controls, with an odds ratio of 1.587 (95% CI: 0.561–4.495; *p* = 0.384). The CC genotype was observed in 32.0% of ACS cases and 34.0% of control subjects, yielding an odds ratio of 1.255 (95% CI: 0.417–3.775; *p* = 0.686). No statistically significant differences were found between groups for any genotype. Hardy–Weinberg equilibrium was maintained in both the ACS (*p* = 0.887) and control (*p* = 0.284) groups. Allele frequency analysis showed a T allele frequency of 43.0% in the ACS group compared to 55.0% in the control group (*p* = 0.089), while the C allele was more common in ACS patients (57.0%) compared to controls (45.0%) (Table 4).

According to Table 5, in the total population, median raftlin levels were 4.27 ng/dL [3.38–5.52] for the TT genotype, 3.79 ng/dL [2.70–5.49] for the TC genotype, and 3.62 ng/dL [2.78–4.99] for the CC genotype, with no statistically significant difference among genotypes (*p* = 0.467). Within the ACS group, raftlin levels were higher across all genotypes, particularly in TT carriers with a median of 4.63 ng/dL [4.18–6.14], followed by CC (4.49 ng/dL [3.69–5.70]) and TC (4.01 ng/dL [2.67–5.98]); however, these differences were not statistically significant (*p* = 0.260). In the control group, raftlin levels were generally lower, with median values of 3.65 ng/dL [2.93–4.51] for TT, 3.47 ng/dL [2.65–5.45] for TC, and 2.90 ng/dL [2.53–3.70] for CC genotypes (*p* = 0.303). No significant association was found between genotype and raftlin concentration in any group (Table 5).

**Figure 1:**
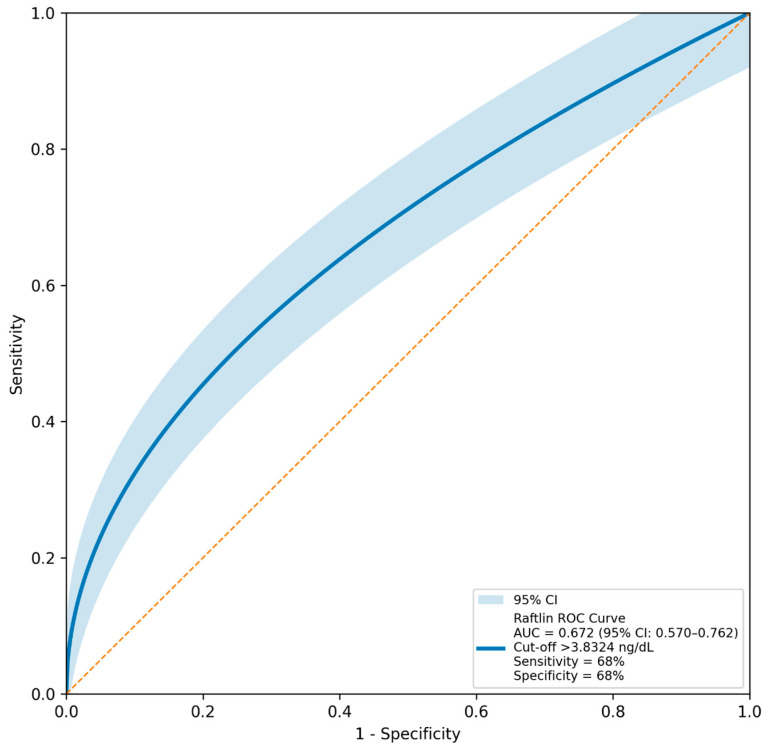
Receiver Operating Characteristic (ROC) Curve Analysis of Raftlin for ACS Detection. ROC curve demonstrating the discriminative performance of serum raftlin levels for identifying acute coronary syndrome (ACS). The area under the curve (AUC) was 0.672 (95% CI: 0.570–0.762; *p* = 0.002). The optimal cut-off value for raftlin was >3.8324 ng/dL, yielding 68% sensitivity and 68% specificity. The shaded area represents the 95% confidence interval. AUC = Area Under the Curve; CI = Confidence Interval.

The ROC curve in Figure 1 illustrates the diagnostic performance of raftlin levels in predicting acute coronary syndrome (ACS). The area under the curve (AUC) was calculated as 0.672 (95% CI: 0.570–0.762), indicating a moderate level of discriminative ability. At the optimal cut-off value of >3.8324 ng/dL, both sensitivity and specificity were calculated as 68%. These findings demonstrate that while raftlin alone may not offer high diagnostic precision, its integration with additional clinical and biochemical markers could potentially improve the accuracy of ACS risk stratification (Figure 1).

According to Table 6, multivariable logistic regression analysis was performed to determine whether the association between raftlin and ACS was independent of baseline clinical differences between groups. The model included raftlin level, left ventricular ejection fraction (LVEF), smoking status, age, and sex. Lower LVEF was identified as an independent predictor of ACS (OR: 0.78, 95% CI: 0.71–0.86, *p* < 0.001). In contrast, although raftlin levels were significantly elevated in ACS patients in univariate analyses, raftlin did not remain independently associated with ACS after adjustment for confounding variables (OR: 1.08, 95% CI: 0.95–1.22, *p* = 0.205). Similarly, smoking status, age, and male sex were not independently associated with ACS in the adjusted model (Table 6).

According to Table 7, additional ROC analyses were performed to compare the diagnostic performance of raftlin with conventional biomarkers used in ACS evaluation. Raftlin demonstrated moderate discriminative ability for ACS detection (AUC: 0.672, 95% CI: 0.570–0.762, *p* = 0.002), while CRP showed a similar diagnostic performance (AUC: 0.639, 95% CI: 0.528–0.739, *p* = 0.018). Troponin and CK-MB demonstrated markedly higher discriminative performance with AUC values of 0.981 and 0.944, respectively (*p* < 0.001 for both). Notably, the combined clinical model integrating raftlin with inflammatory, metabolic, and clinical variables demonstrated substantially improved discriminative performance (AUC: 0.901, 95% CI: 0.833–0.949, *p* < 0.001) compared with raftlin alone (Table 7).

**Figure 2:**
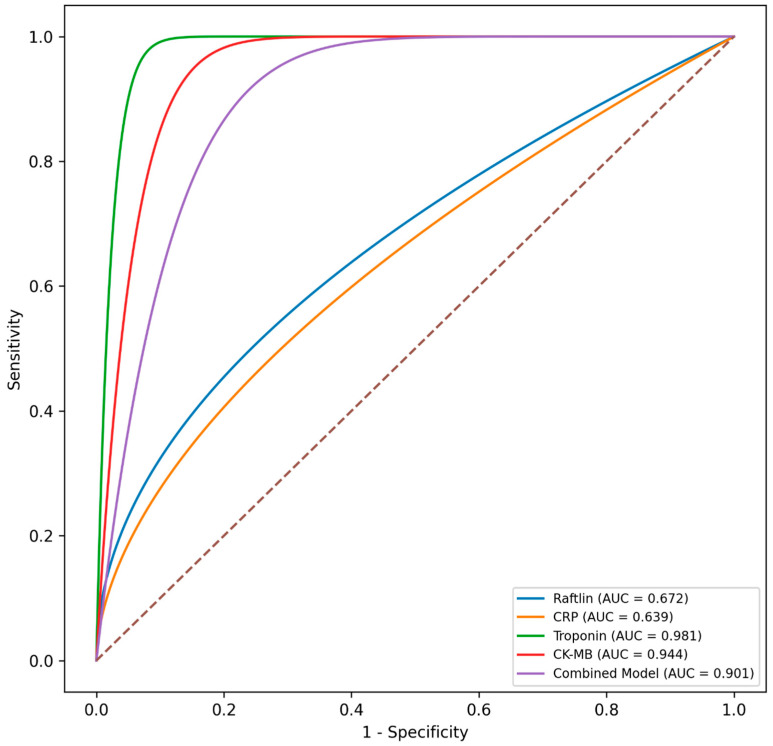
Comparative ROC Curve Analysis of Raftlin and Conventional Biomarkers for ACS Detection. Receiver operating characteristic (ROC) curves comparing the diagnostic performances of raftlin, CRP, troponin, CK-MB, and the combined clinical model for the detection of acute coronary syndrome (ACS). The combined model demonstrated superior discriminative performance compared with raftlin alone. AUC = Area Under the Curve; CRP = C-Reactive Protein; CK-MB = Creatine Kinase-MB.

The comparative ROC analyses presented in Figure 2 demonstrated that raftlin alone showed moderate discriminative ability for ACS detection (AUC: 0.672), whereas conventional biomarkers such as troponin and CK-MB demonstrated substantially higher diagnostic performance. Notably, the combined clinical model integrating raftlin with inflammatory, metabolic, and clinical parameters yielded markedly improved discriminative performance (AUC: 0.901), supporting the potential value of multimarker approaches in ACS risk assessment (Figure 2).

According to Table 8, net reclassification improvement (NRI) analysis was performed to evaluate whether the addition of raftlin improved risk classification beyond the baseline clinical model. The incorporation of raftlin into the baseline model resulted in a modest but statistically significant improvement in risk reclassification for ACS prediction (NRI: 0.213, *p* = 0.041). However, bootstrap resampling and 5-fold cross-validation analyses demonstrated only minimal changes in overall model discrimination after the addition of raftlin. These findings suggest that while raftlin may provide limited complementary reclassification information when integrated with conventional inflammatory and clinical parameters, its incremental contribution to overall predictive performance should be interpreted cautiously because of the exploratory nature and relatively limited sample size of the study (Table 8).

## 4. Discussion

The principal finding of the present study was that circulating raftlin levels were significantly elevated in patients with acute coronary syndrome compared with healthy controls and were associated with metabolic and inflammatory parameters such as LDL and HbA1c. However, despite this significant association, raftlin demonstrated only moderate diagnostic performance and did not remain an independent predictor of ACS after adjustment for baseline clinical variables including left ventricular ejection fraction. These findings suggest that raftlin may reflect the broader inflammatory and metabolic dysregulation accompanying ACS rather than serving as a disease-specific standalone diagnostic biomarker.

Despite substantial advances in diagnostic and therapeutic strategies, acute coronary syndrome (ACS) continues to be associated with considerable morbidity and mortality. Since inflammation plays a central role in plaque destabilization and acute coronary events, numerous inflammatory biomarkers have been investigated in ACS, including conventional markers such as C-reactive protein (CRP). In this context, the present study aimed to explore whether raftlin, a lipid raft-associated inflammatory protein, may provide additional insight into the inflammatory background of ACS. Importantly, this study was not designed to perform a head-to-head ROC comparison between raftlin and CRP. Therefore, the superiority of raftlin over conventional inflammatory biomarkers cannot be claimed based on the current analyses. Given its moderate AUC, raftlin is more plausibly positioned as one component of a multimarker inflammatory profile rather than as a standalone diagnostic biomarker. It should also be considered that the control cohort did not consist of completely healthy community-based volunteers but rather of individuals with angiographically normal coronary arteries who had undergone cardiovascular evaluation because of clinical suspicion or symptoms. Although obstructive epicardial coronary artery disease was excluded, the potential presence of coronary microvascular dysfunction, endothelial inflammatory activity, or subclinical vascular disease cannot be entirely ruled out in these individuals. This selection characteristic may have reduced the biological contrast between groups and consequently attenuated the observed discriminative performance of raftlin, including the estimated AUC values. Therefore, the moderate diagnostic performance observed in the present study should be interpreted within the context of this potential selection bias. Accordingly, the present study should not be interpreted as demonstrating superiority of raftlin over established ACS biomarkers such as cardiac troponins. Rather, the potential value of raftlin may lie in its ability to reflect inflammatory and metabolic activation accompanying acute coronary events. In this context, raftlin may provide complementary pathophysiological information beyond conventional myocardial necrosis markers, particularly within multimarker inflammatory models. Therefore, although the present findings do not support the routine standalone clinical use of raftlin for ACS diagnosis, they contribute exploratory evidence regarding the relationship between lipid raft-associated inflammatory signaling and acute coronary syndromes. In this regard, raftlin may provide complementary information regarding inflammatory and metabolic activation accompanying ACS rather than replacing established biomarkers with high diagnostic performance, such as cardiac troponins. In the present study, the combined clinical model incorporating raftlin together with conventional inflammatory, metabolic, and clinical variables demonstrated substantially improved discriminative performance compared with raftlin alone. Consistent with this observation, net reclassification improvement (NRI) analysis demonstrated a modest but statistically significant improvement in ACS risk classification after the addition of raftlin to the baseline clinical model. Although the incremental contribution was limited, this finding further supports the concept that raftlin may provide complementary pathophysiological information when interpreted together with established inflammatory and clinical markers. The predominant mechanism implicated in ACS pathophysiology is atherosclerosis—a chronic inflammatory condition characterized by endothelial dysfunction, subendothelial lipid accumulation, smooth muscle cell proliferation, apoptosis, necrosis, and both local and systemic inflammation. This process heavily involves inflammatory molecules and lipids, which are considered the two key elements driving disease progression. Recent evidence has further emphasized the complex interaction between vascular inflammation, lipid raft integrity, endocrine signaling pathways, and plaque vulnerability in atherosclerosis. In particular, the GH–IGF-1 axis and S-Klotho have been implicated in endothelial dysfunction, inflammatory plaque phenotypes, oxidative stress regulation, and vascular immune modulation, all of which contribute to plaque destabilization and atherosclerotic progression [11]. Given the established role of raftlin as a lipid raft-associated inflammatory protein, these mechanisms may provide additional biological context linking lipid raft signaling pathways with inflammatory plaque vulnerability in ACS. Studies have shown that white blood cell infiltration into plaques can accelerate coronary artery stenosis over time [12]. Our findings are consistent with this inflammatory mechanism, as elevated raftlin levels—a marker associated with leukocyte activation and trafficking—were observed in ACS patients, suggesting that raftlin may reflect heightened immune cell activity contributing to plaque instability. Importantly, exclusion of patients with chronic inflammatory diseases was not intended to negate the role of chronic inflammation in atherosclerosis or ACS pathophysiology. Rather, this exclusion strategy was applied to minimize the influence of systemic inflammatory disorders that could independently and substantially alter circulating inflammatory biomarker levels beyond the vascular inflammatory processes directly related to atherosclerosis and ACS. In this context, the present study focused primarily on inflammation associated with coronary atherosclerotic disease itself rather than inflammation secondary to major systemic immune-mediated disorders. It should be emphasized that raftlin is not disease-specific and may be elevated in various inflammatory or immune-mediated conditions. Accordingly, in ACS, raftlin should be interpreted as a supportive inflammatory signal reflecting immune activation rather than a definitive disease-specific diagnostic marker.

Numerous studies have investigated the relationship between ACS and inflammation. One study demonstrated the presence of macrophages and T cells in coronary plaques, highlighting their role in inflammatory processes that weaken plaque structure and contribute to the development of ACS [13,14]. In our study, raftlin levels were positively correlated with LDL (r = 0.298, *p* = 0.036) and HbA1c (r = 0.335, *p* = 0.017) in ACS patients, indicating a significant relationship between lipid metabolism, glycemic control, and the inflammatory processes that contribute to the weakening of plaques in ACS, as discussed by previous studies. This pattern may reflect a metabolic–inflammatory interface in ACS, where dyslipidemia and impaired glycemic control contribute to immune activation and vascular inflammation. Although mechanistic pathways were not evaluated in this study, raftlin could represent a downstream signal of inflammatory activation driven by metabolic stress, rather than a direct causal mediator; targeted experimental studies are needed to clarify this possibility. The shift in the balance between fibroblasts and macrophages towards macrophages in coronary plaques accelerates plaque degradation, weakens structural integrity, and predisposes plaques to rupture. This inflammatory shift aligns with the observed correlation between raftlin and LDL/HbA1c in our study, suggesting that raftlin may mirror the inflammatory milieu promoting this cellular imbalance. Sager et al. emphasized the involvement of inflammation at every stage of ACS development [15]. In our study, raftlin levels were significantly higher in the ACS group (mean: 4.28 ng/dL) compared to the control group (mean: 3.38 ng/dL; *p* = 0.003). This elevation further supports the role of inflammation in the pathogenesis of ACS, aligning with the findings of Sager et al. regarding the critical role of inflammation in ACS development. Similarly, Bentzon et al. demonstrated that coronary plaque rupture is secondary to inflammation-induced weakening of the fibrous cap [16]. The present findings demonstrated that circulating raftlin levels were significantly elevated in patients with ACS compared to controls. However, the present study evaluated ACS as a unified clinical entity and did not investigate potential differences among STEMI, NSTEMI, and unstable angina subgroups. Given the known heterogeneity of inflammatory burden across ACS phenotypes, the observed raftlin elevations should be interpreted as reflecting overall inflammatory activation associated with ACS rather than subtype-specific biological behavior. However, after adjustment for important baseline variables including left ventricular ejection fraction, smoking status, age, and sex, raftlin did not remain an independent predictor of ACS. Smoking prevalence was also significantly higher in the ACS group and may have contributed to the increased inflammatory burden observed in these patients. However, smoking status was included in the multivariable regression model and was not independently associated with ACS after adjustment. This observation suggests that increased raftlin levels may partly reflect the broader inflammatory and cardiovascular dysfunction profile accompanying ACS rather than representing a fully disease-specific and independent biomarker. In particular, the significantly lower LVEF observed in the ACS group may have contributed to the heightened inflammatory milieu, since impaired ventricular systolic function itself is closely associated with systemic inflammation and endothelial dysfunction. However, the present study evaluated ventricular function primarily through global left ventricular ejection fraction and did not include detailed echocardiographic parameters such as regional wall motion abnormalities, diastolic dysfunction indices, or segmental systolic function analyses. These echocardiographic characteristics may provide additional insight into the relationship between myocardial dysfunction, inflammatory activation, and circulating raftlin levels in ACS. Therefore, the observed association between reduced LVEF and ACS should be interpreted within the limitations of the available echocardiographic dataset. Therefore, while raftlin appears to be associated with inflammatory activation in ACS, its diagnostic utility may be more appropriate as a complementary biomarker integrated into multimarker inflammatory models rather than as a standalone predictor. In this regard, it may be argued that the pathophysiological role of raftlin could potentially be more relevant in chronic inflammatory states associated with accelerated atherosclerosis progression rather than in the acute diagnostic setting of ACS alone. Chronic inflammatory disorders such as rheumatoid arthritis, systemic autoimmune diseases, and chronic renal dysfunction are characterized by persistent vascular inflammation and endothelial activation, which may provide a more suitable biological context for investigating the long-term relationship between raftlin signaling and atherosclerotic disease progression. Accordingly, the present findings should be interpreted primarily as exploratory observations regarding inflammatory activation during ACS, whereas future longitudinal studies involving chronic inflammatory populations may better clarify whether raftlin participates in the progression of atherosclerosis and subsequent coronary artery disease development.

To our knowledge, limited data are available regarding the association between acute coronary syndrome and circulating raftlin levels. In this context, the present study contributes preliminary clinical data suggesting a possible relationship between raftlin and inflammatory processes accompanying ACS. However, given the moderate diagnostic performance observed and the limited sample size, the present findings should be considered exploratory rather than definitive. Further large-scale and longitudinal studies are required to clarify whether raftlin has meaningful clinical or translational utility in ACS. Consistent with our findings, Belce et al. reported significantly elevated raftlin levels in patients with atherosclerotic cardiovascular disease, along with a substantial decrease following statin therapy, supporting the link between raftlin expression and vascular inflammation. This supports raftlin’s potential as a treatment-responsive biomarker of vascular inflammation [17]. While Belce et al. reported decreased raftlin levels post-statin therapy, our study reinforces raftlin’s diagnostic relevance in untreated ACS patients. This suggests that raftlin may serve both as a marker of active inflammation and a treatment-response biomarker in longitudinal monitoring. In our analysis, raftlin levels were significantly higher in the ACS group (mean: 4.28 ng/dL) compared to the control group (mean: 3.38 ng/dL; *p* = 0.003). Statins have been demonstrated to reduce the inflammatory biomarkers in atherosclerotic plaques, which aligns with the reduced raftlin levels observed post-treatment.

A study by Belce et al. compared oxidative stress and inflammatory biomarkers before and after treatment in patients with atherosclerotic cardiovascular disease. Statin therapy was initiated in the patient group, and comparisons were made between pre- and post-treatment values as well as between the patient and control groups. Raftlin levels were significantly higher in the patient group compared to controls and showed a significant reduction after statin therapy [17]. These findings suggest a strong link between raftlin levels, inflammation, and atherosclerosis. Our findings align with the study by Belce et al., where raftlin levels were significantly higher in patients with atherosclerotic cardiovascular disease, and these levels decreased significantly following statin therapy. Nevertheless, a direct comparison with Belce et al. is limited because our study did not standardize or longitudinally assess statin exposure at the time of blood sampling, whereas Belce et al. specifically evaluated pre- and post-statin changes. Differences in study populations (stable atherosclerotic disease vs. acute presentation), timing of sampling, and treatment status may explain apparent discrepancies across studies and should be addressed in future longitudinal designs.

Our study also investigated the relationship between raftlin levels, ACS, and RFTN1 gene polymorphism (rs690037). While raftlin levels were significantly higher in the ACS group, no significant differences were found in the distribution of TT, TC, and CC genotypes between the ACS and control groups (*p* > 0.05). Additionally, no significant relationship was observed between raftlin levels and genotypes in either group (*p* > 0.05).

To our knowledge, no prior studies have explored the association between rs690037 polymorphism and ACS. However, a study examining the relationship between raftlin levels and rs690037 polymorphism in primary open-angle glaucoma found no significant correlation between raftlin levels and genotypic variations [17]. Similarly, our study found no significant association between the rs690037 polymorphism and raftlin levels in both the ACS and control groups (*p* > 0.05), suggesting that raftlin regulation in ACS may be influenced by factors other than genetic variations, as previously observed in primary open-angle glaucoma by Chen et al. [18]. This mirrors the findings by Chen et al. in primary open-angle glaucoma, suggesting that raftlin expression may be modulated more by environmental or post-transcriptional mechanisms than by genetic polymorphisms alone across different disease models. Such factors may include post-transcriptional regulation, microRNA activity, or epigenetic modifications—none of which were evaluated in the current study but warrant further investigation. The findings of our study contribute novel insights to the existing literature, providing a basis for future research into the role of raftlin and its genetic determinants in cardiovascular diseases.

**Limitations of the Study**

This study has several limitations that should be considered. First, although an a priori sample size calculation was performed, the study was conducted at a single center with a relatively small cohort (50 ACS patients and 50 controls), which may limit the precision of diagnostic estimates, genetic associations, and the generalizability of the findings to broader populations. Furthermore, the relatively limited sample size may have increased the susceptibility of multivariable analyses to model overfitting despite the restricted number of variables included in the adjusted models. Therefore, the regression and reclassification findings should be interpreted cautiously and considered exploratory rather than definitive. In addition, the present study did not include longitudinal or prognostic outcome data; consequently, the potential value of raftlin for long-term cardiovascular risk prediction could not be evaluated. Another important limitation is the heterogeneity of the ACS population, which included patients with STEMI, NSTEMI, and unstable angina. These clinical entities differ substantially in terms of myocardial injury burden, inflammatory activation, plaque morphology, and thrombotic activity. Therefore, pooling all ACS subtypes into a single analysis may have obscured clinically meaningful subgroup-specific differences in circulating raftlin levels and inflammatory responses. Because of the relatively limited sample size, separate subgroup analyses for STEMI, NSTEMI, and unstable angina were not performed. Future studies involving larger ACS cohorts are needed to determine whether raftlin demonstrates differential behavior across distinct ACS phenotypes. This clinical heterogeneity may have contributed to variability in inflammatory responses and biomarker expression patterns. Moreover, although blood samples were collected during the initial clinical evaluation whenever feasible, exact sampling times could not be completely standardized across all patients. Another important limitation is the absence of standardized assessment of preexisting statin and antiplatelet therapy exposure at the time of blood sampling. Statins are known to exert substantial anti-inflammatory and endothelial-modulating effects and may influence circulating inflammatory biomarker levels, including raftlin-related signaling pathways. Similarly, antiplatelet therapies may alter inflammatory activation during acute coronary events. Therefore, variability in baseline cardiovascular pharmacotherapy may have acted as an important confounding factor and could have influenced both circulating raftlin concentrations and the observed diagnostic performance estimates. Finally, while circulating raftlin levels were significantly elevated in ACS patients, raftlin demonstrated only moderate discriminative performance and lost independent significance after multivariable adjustment, suggesting that raftlin may primarily reflect nonspecific inflammatory activation rather than serving as a disease-specific standalone biomarker for ACS. Multicenter studies with larger sample sizes could provide more robust and externally valid results. Additionally, the control group consisted of individuals undergoing cardiovascular evaluation with angiographically normal coronary arteries rather than asymptomatic community-based healthy volunteers. Therefore, selection bias cannot be completely excluded.

Second, medication status—particularly statin exposure and the timing of blood sampling relative to medical treatment—was not standardized. This may have influenced circulating raftlin levels and limited direct comparison with longitudinal treatment studies evaluating inflammatory biomarker dynamics.

Third, although comparative ROC analyses with conventional biomarkers were performed, the combined predictive model and NRI analysis were derived from a relatively limited single-center cohort and were not externally validated. Therefore, the incremental diagnostic contribution of raftlin should be interpreted cautiously until confirmed in independent populations.

Fourth, the long-term prognostic significance of elevated raftlin levels in ACS patients was not assessed. Studies with extended follow-up periods are needed to clarify whether raftlin has predictive value for adverse cardiovascular outcomes.

Finally, while multiple statistical tests were applied, no formal correction for multiple comparisons (e.g., Bonferroni adjustment) was performed, which may have increased the risk of type I error. Therefore, statistically significant findings—particularly weak correlation analyses—should be interpreted cautiously until validated in larger cohorts.

## 5. Conclusions

This study demonstrated that circulating raftlin levels were elevated in patients with acute coronary syndrome and were weakly associated with certain metabolic and inflammatory parameters, including LDL and HbA1c. Although raftlin showed moderate discriminative performance for ACS detection, it did not remain an independent predictor after adjustment for important clinical confounders. These findings suggest that raftlin may reflect the broader inflammatory and metabolic dysregulation accompanying ACS rather than serving as a disease-specific standalone biomarker.

Furthermore, combined predictive models incorporating raftlin together with conventional inflammatory and clinical parameters demonstrated improved discriminative performance compared with raftlin alone, supporting the potential value of multimarker approaches in ACS risk assessment. Nevertheless, given the limited sample size, observational design, and lack of external validation, these findings should be interpreted cautiously and considered exploratory. Larger prospective and longitudinal studies are required to clarify the potential incremental clinical value of raftlin in ACS.

## Figures and Tables

**Table 1:** Demographic and Clinical Characteristics of ACS and Control Groups (*n* = 100).

Demographic and Clinical Features	ACS Group (*n* = 50)	Control Group (*n* = 50)	*p*-Value
Age (Mean ± SD)	58.20 ± 6.94	57.40 ± 5.52	0.525
Gender, Female (%)	24 (48%)	26 (52%)	0.689
Gender, Male (%)	26 (52%)	24 (48%)	—
BMI (Mean ± SD)	27.41 ± 3.95	28.39 ± 3.53	0.198
EF (Median [IQR])	50 [40–55]	60 [60–60]	**<0.0001**
DM (%)	17 (34%)	14 (28%)	0.517
HT (%)	21 (42%)	29 (58%)	0.110
HL (%)	11 (22%)	11 (22%)	1.00
Smoking (%)	35 (70%)	23 (46%)	**0.015**
Raftlin (Median [IQR])	4.28 [3.48–5.78]	3.38 [2.66–4.23]	**0.003**

Statistical analyses included Student’s *t*-test for continuous variables with normal distribution, the Mann–Whitney U test for continuous variables without normal distribution, and the Chi-square test for categorical data. Statistically significant results are highlighted in bold within the table. A *p*-value below 0.05 was accepted as the threshold for statistical significance. Abbreviations: ACS, Acute Coronary Syndrome; BMI, Body Mass Index; EF, Ejection Fraction; DM, Diabetes Mellitus; HT, Hypertension; HL, Hyperlipidemia; IQR, Interquartile Range; SD, Standard Deviation.

**Table 2:** Correlation Between Raftlin Levels and Clinical Parameters (*n* = 100).

Variables	All Population (*n* = 100)		ACS Group (*n* = 50)		Control Group (*n* = 50)	
	r-Value	*p*-Value	r-Value	*p*-Value	r-Value	*p*-Value
Age	0.117	0.245	0.191	0.185	−0.038	0.793
BMI	−0.116	0.252	−0.031	0.832	−0.205	0.154
Urea	−0.052	0.608	−0.059	0.684	−0.162	0.262
Creatinine	0.126	0.212	0.271	0.057	−0.057	0.692
GFR	−0.112	0.267	−0.100	0.489	−0.067	0.646
Sodium	−0.072	0.475	−0.010	0.948	0.095	0.514
Potassium	−0.184	0.067	−0.205	0.153	−0.005	0.973
Uric Acid	0.085	0.399	0.123	0.396	0.042	0.770
**LDL**	**0.223**	**0.025**	**0.298**	**0.036**	0.066	0.647
HDL	0.123	0.222	0.182	0.206	0.143	0.323
TG	−0.065	0.523	−0.121	0.404	0.015	0.918
BNP	0.094	0.350	0.024	0.868	0.177	0.220
**HbA1c**	**0.201**	**0.045**	**0.335**	**0.017**	−0.132	0.361
Troponin	0.010	0.919	−0.169	0.242	0.042	0.774
CK-MB	0.019	0.847	−0.120	0.406	0.002	0.990
LVEF	−0.045	0.658	0.066	0.648	0.016	0.912
CRP	0.145	0.149	0.118	0.416	0.071	0.625

Correlation coefficients (r-values) were calculated to examine relationships between raftlin levels and clinical variables. Correlation analyses were performed using Pearson correlation coefficients for variables with normal distribution and Spearman correlation coefficients for variables without normal distribution. Statistically significant *p*-values (*p* < 0.05) are indicated in bold in the table, while values below 0.001 are presented as *p* < 0.001. Abbreviations: LDL, Low-Density Lipoprotein; HbA1c, Glycated Hemoglobin; BMI, Body Mass Index; GFR, Glomerular Filtration Rate; BNP, B-type Natriuretic Peptide; TG, Triglycerides; HDL, High-Density Lipoprotein; LVEF, Left Ventricular Ejection Fraction; CRP, C-Reactive Protein.

**Table 3:** ROC Analysis of Raftlin Levels for ACS Detection (*n* = 100).

Parameter	AUC [CI]	*p*-Value	Cut-Off	Sensitivity [CI]	Specificity [CI]
Raftlin	0.672 [0.570–0.762]	**0.0020**	>3.8324	68 [53.3–80.5]	68 [53.3–80.5]

Receiver Operating Characteristic (ROC) curve analysis was used to assess the ability of raftlin concentrations to discriminate between patients with ACS and control subjects. The area under the curve (AUC) quantifies the overall diagnostic performance, with an AUC value of 1.0 indicating perfect discrimination. The sensitivity and specificity at the determined cut-off value (>3.8324) were calculated to assess the test’s diagnostic utility. In the table, statistically significant *p*-values (*p* < 0.05) are marked in bold. AUC = Area Under the Curve, CI = Confidence Interval.

**Table 4:** Genotype Distribution in ACS and Control Groups (*n* = 100).

Genotype	ACS Group (*n* = 50)	Control Group (*n* = 50)	Odds Ratio [95% CI]	*p*-Value
TT	9 (18.0%)	12 (24.0%)	Ref.	0.668
TC	25 (50.0%)	21 (42.0%)	1.587 [0.561–4.495]	0.384
CC	16 (32.0%)	17 (34.0%)	1.255 [0.417–3.775]	0.686

The Hardy–Weinberg equilibrium test was used to assess whether the genotype distributions in both the ACS and control groups were consistent with expected proportions. Odds ratios (ORs) and 95% confidence intervals (CIs) were calculated to determine the likelihood of TC and CC genotypes in the ACS group compared to the control group. *p*-values were derived from Chi-square tests. In the table, no statistically significant differences were observed. CI = Confidence Interval, Ref. = Reference Category.

**Table 5:** Raftlin Levels by Genotype in Total Population, ACS Group, and Control Group (*n* = 100).

Genotype	Total Population (*n* = 100) Median [Q1–Q3]	ACS Group (*n* = 50) Median [Q1–Q3]	Control Group (*n* = 50) Median [Q1–Q3]	*p*-Value
TT	4.27 [3.38–5.52]	4.63 [4.18–6.14]	3.65 [2.93–4.51]	0.467
TC	3.79 [2.70–5.49]	4.01 [2.67–5.98]	3.47 [2.65–5.45]	0.260
CC	3.62 [2.78–4.99]	4.49 [3.69–5.70]	2.90 [2.53–3.70]	0.303

The Mann–Whitney U test was used to evaluate differences in raftlin levels across genotypes in the total population, ACS group, and control group. Median and interquartile range (Q1–Q3) were calculated for raftlin levels, and *p*-values were used to assess statistical significance. In the table, no statistically significant differences were observed. ACS = Acute Coronary Syndrome, Q1–Q3 = Interquartile Range.

**Table 6:** Multivariable Logistic Regression Analysis for the Presence of Acute Coronary Syndrome.

Variable	Odds Ratio (OR)	95% Confidence Interval	*p*-Value
Raftlin	1.08	0.95–1.22	0.205
LVEF	0.78	0.71–0.86	**<0.001**
Smoking	1.64	0.71–3.78	0.245
Age	1.02	0.95–1.10	0.511
Male sex	1.21	0.53–2.76	0.648

Data are presented as odds ratio (OR) with 95% confidence interval (CI). Multivariable logistic regression analysis was performed to identify independent predictors of acute coronary syndrome (ACS). Variables included in the model were selected based on baseline clinical relevance and significant intergroup differences. LVEF: Left ventricular ejection fraction. Statistically significant *p*-values are shown in bold.

**Table 7:** Comparative ROC Analysis of Raftlin and Conventional Biomarkers for ACS Detection (*n* = 100).

Parameter	AUC	95% CI	*p*-Value
Raftlin	0.672	0.570–0.762	**0.002**
CRP	0.639	0.528–0.739	**0.018**
Troponin	0.981	0.943–0.998	**<0.001**
CK-MB	0.944	0.878–0.980	**<0.001**
Combined clinical model *	0.901	0.833–0.949	**<0.001**

Receiver operating characteristic (ROC) curve analyses were performed to compare the discriminative performances of raftlin and conventional biomarkers for the detection of acute coronary syndrome (ACS). The combined clinical model included raftlin, CRP, LDL, HbA1c, LVEF, smoking status, age, and sex. Area under the curve (AUC) values with 95% confidence intervals (CI) are presented. Statistically significant *p*-values are shown in bold. Abbreviations: AUC, Area Under the Curve; CI, Confidence Interval; CRP, C-Reactive Protein; CK-MB, Creatine Kinase-MB; * indicates that the combined clinical model included raftlin, CRP, LDL cholesterol, HbA1c, LVEF, smoking status, age, and sex.

**Table 8:** Net Reclassification Improvement (NRI) Analysis After Addition of Raftlin to the Baseline Clinical Model (*n* = 100).

Model Comparison/Validation Metric	Result
Baseline clinical model AUC	0.901
Baseline clinical model + raftlin AUC	0.904
ΔAUC after addition of raftlin	0.003
Bootstrap 95% CI for ΔAUC	−0.003 to 0.031
Bootstrap *p*-value for ΔAUC	0.508
NRI after addition of raftlin	0.213
NRI *p*-value	0.041
Optimism-corrected AUC, baseline clinical model	0.879
Optimism-corrected AUC, baseline clinical model + raftlin	0.877
5-fold cross-validated AUC, baseline clinical model	0.863
5-fold cross-validated AUC, baseline clinical model + raftlin	0.865

Net reclassification improvement (NRI) analysis was performed to evaluate the incremental contribution of raftlin beyond the baseline clinical model for ACS prediction. The baseline clinical model included CRP, LDL cholesterol, HbA1c, left ventricular ejection fraction (LVEF), smoking status, age, and sex. Positive NRI values indicate improved risk classification after the addition of raftlin. To further assess model stability and potential optimism, bootstrap resampling and 5-fold cross-validation analyses were additionally performed for both the baseline clinical model and the baseline clinical model plus raftlin. Abbreviations: NRI, Net Reclassification Improvement.

## Data Availability

The data supporting the findings of this study are available from the corresponding author upon reasonable request. Due to institutional and ethical regulations, the raw data are not publicly available.

## Abbreviations

ACS	Acute Coronary Syndrome
AUC	Area Under the Curve
BMI	Body Mass Index
BNP	B-type Natriuretic Peptide
CC	Creatine Kinase-MB
CI	Confidence Interval
CK-MB	Creatine Kinase-MB
CRP	C-Reactive Protein
DM	Diabetes Mellitus
DNA	Deoxyribonucleic Acid
EF	Ejection Fraction
ELISA	Enzyme-Linked Immunosorbent Assay
GFR	Glomerular Filtration Rate
HbA1c	Glycated Hemoglobin
HDL	High-Density Lipoprotein
HL	Hyperlipidemia
HT	Hypertension
IQR	Interquartile Range
LDL	Low-Density Lipoprotein
LVEF	Left Ventricular Ejection Fraction
NF-κB	Nuclear Factor Kappa B
NRI	Net Reclassification Improvement
NSTEMI	Non-ST-Segment Elevation Myocardial Infarction
OD	Optical Density
OR	Odds Ratio
PCR	Polymerase Chain Reaction
ROC	Receiver Operating Characteristic
RFTN1	Raftlin Family Member 1 Gene
SD	Standard Deviation
STEMI	ST-Segment Elevation Myocardial Infarction
TG	Triglycerides
WHO	World Health Organization
